# Determinants of sphenoid sinus ostium localization: implications for safer endoscopic sinus surgery

**DOI:** 10.1007/s00405-026-10065-7

**Published:** 2026-03-09

**Authors:** Murat Golpinar, Gurbet Yanarates, Erdal Komut, Hande Salim Ari, Gulcin Aydogdu, Figen Govsa

**Affiliations:** 1 Faculty of Medicine, Department of Anatomy, Hitit University, Corum, Turkey; 2 Faculty of Medicine, Department of Radiology, Hitit University, Corum, Turkey; 3 Faculty of Medicine, Department of Biostatistics, Hitit University, Corum, Turkey; 4 Faculty of Medicine, Department of Anatomy, Ege University, Izmir, Turkey

**Keywords:** Sphenoid sinus, Rostrum pneumatization, Onodi cells, Sphenoid sinus ostium, Endoscopic sinus surgery, Computed tomography

## Abstract

**Purpose:**

This study aimed to evaluate multiple anatomical and morphological factors influencing the localization of the sphenoid sinus ostium (SSO) to improve surgical safety in endoscopic sinus surgery.

**Methods:**

Computed tomography (CT) images of 302 subjects were examined. The vertical and horizontal positions of the SSO were assessed using standardized reference lines on sagittal and axial sections. Pneumatization patterns were classified on sagittal and coronal planes. Rostrum pneumatization and the presence of Onodi cells were also evaluated. Morphometric measurements and potential influencing factors were analyzed using logistic and linear regression models.

**Results:**

The SSO was most frequently located in the superior (72.8%) and medial (55.6%) thirds. The female gender increased the likelihood of medial ostium localization by 2.715-fold. Compared to the sellar type, conchal pneumatization increased the probability of medial positioning by 9.82-fold, while the postsellar type reduced it (OR: 0.208). The postrotundum type was strongly associated with superior and medial localizations (OR: 3.289 and OR: 2.978, respectively). A wider sphenoid rostrum reduced the probability of medial localization, whereas rostrum pneumatization promoted superior displacement of the SSO. Onodi cells and increased sinus height shifted the SSO inferiorly by 1.929 and 0.341 units, respectively.

**Conclusions:**

The localization of the SSO was found to be significantly influenced by gender, sphenoid sinus pneumatization patterns, rostrum morphology, and the presence of Onodi cells. Careful preoperative evaluation of CT images for these factors may provide valuable insights for surgeons and help minimize the risk of serious complications during endoscopic sinus surgery.

## Introduction

The sphenoid sinuses (SS) are bilaterally paired, irregularly shaped cavities located within the sphenoid bone at the center of the cranial base. They are unique due to their highly variable morphology, pneumatization, and proximity to important neurovascular structures, such as the internal carotid artery, optic nerve, cavernous sinus, and pituitary gland. Their anatomical proximity to these vital structures makes them a critical region of the skull base in various neurosurgical interventions, including transsphenoidal procedures. The SS also provides a natural surgical corridor for the treatment of skull base pathologies in adjacent sellar regions [1, 2]. However, their close relationship with multiple neurovascular structures, together with their variable morphology and pneumatization, may give rise to serious complications during surgery [2, 3].

The sphenoid sinus ostium (SSO) is the anterior opening of the sinus, which communicates directly with the sphenoethmoidal recess. It is located inferior to the middle cranial fossa, medial to the superior turbinate, and lateral to the nasal septum. The SSO is considered an important surgical landmark because it provides a safe and direct route to the SS without injuring adjacent structures [4]. The vertical and horizontal distances of the SSO from key reference points supply essential information for the determination of the precise localization, thereby facilitating safe surgical access to the SS [5]. Although the SSO is generally located in the middle portion of the anterior sinus wall, both its vertical and horizontal positions may show considerable variation [6–8]. As a result, identifying the exact localization of the SSO is not always straightforward during surgical interventions on the SS. Various pneumatization patterns and surrounding bony structures of the SS can significantly influence the position of this opening [3, 9]. A thorough understanding of the morphological variations of the SS and the position and anatomic features of the SSO is essential to be able to determine the safest surgical corridor during transsphenoidal procedures. Nevertheless, there is a limited number of studies in the literature that have investigated the potential impact of morphological variations and adjacent structures of the SS on the localization of the SSO.

The aim of this study was to investigate the localization of the SSO with respect to important anatomic landmarks and to analyze the influence of morphological characteristics and adjacent structures of the SS on both the vertical and horizontal position of the SSO.

## Materials and methods

### Subjects

Approval for this study was granted by the Ethics and Research Committee of Hitit University (protocol number: 2022/17). Paranasal sinus computed tomography (PNCT) images of 302 patients (150 males and 152 females), aged 18–82 years, who met the inclusion criteria and visited Erol Olçok Training and Research Hospital between April 2016 and June 2021, were retrospectively retrieved from the Radiology Department of Hitit University. The inclusion criteria comprised adult individuals (≥ 18 years) with fully developed sphenoid sinuses and high-resolution paranasal sinus CT scans that provided clear visualization of the sphenoid sinus, the SSO, and the surrounding anatomical structures. Exclusion criteria comprised individuals younger than 18 years and adults with a history of sinonasal or skull base surgery, trauma, pathological conditions, or congenital anomalies that could affect sphenoid sinus development, as well as those with any radiological evidence of sinonasal pathology. CT images that were affected by motion artifacts, had inadequate resolution, or could not provide complete visualization of the sphenoid sinus region and adjacent structures were excluded.

### Paranasal sinus computed tomography images analysis

Image analysis was conducted using a 128-slice Optima CT660 scanner (General Electric Medical Systems, Milwaukee, WI, USA). All scans were obtained with the patient in the supine position, without the use of sedation or contrast media. Axial sections were acquired with the following imaging parameters: slice thickness of 1.50 mm, interslice interval of 1.0 mm, 100 kV, 120 mA, pitch of 0.531, rotation time of 0.6 s, collimation of 20 mm, matrix size of 512 × 512, and a field of view (FOV) of 20 cm. Coronal reconstructions were generated from the axial images using the Volume Viewer software (General Electric Medical Systems, Milwaukee, WI, USA), with a reconstructed slice thickness of 1.00 mm and an interval of 1.25 mm.

### Morphological characteristics of the sphenoid sinus ostium

**Fig. 1:**
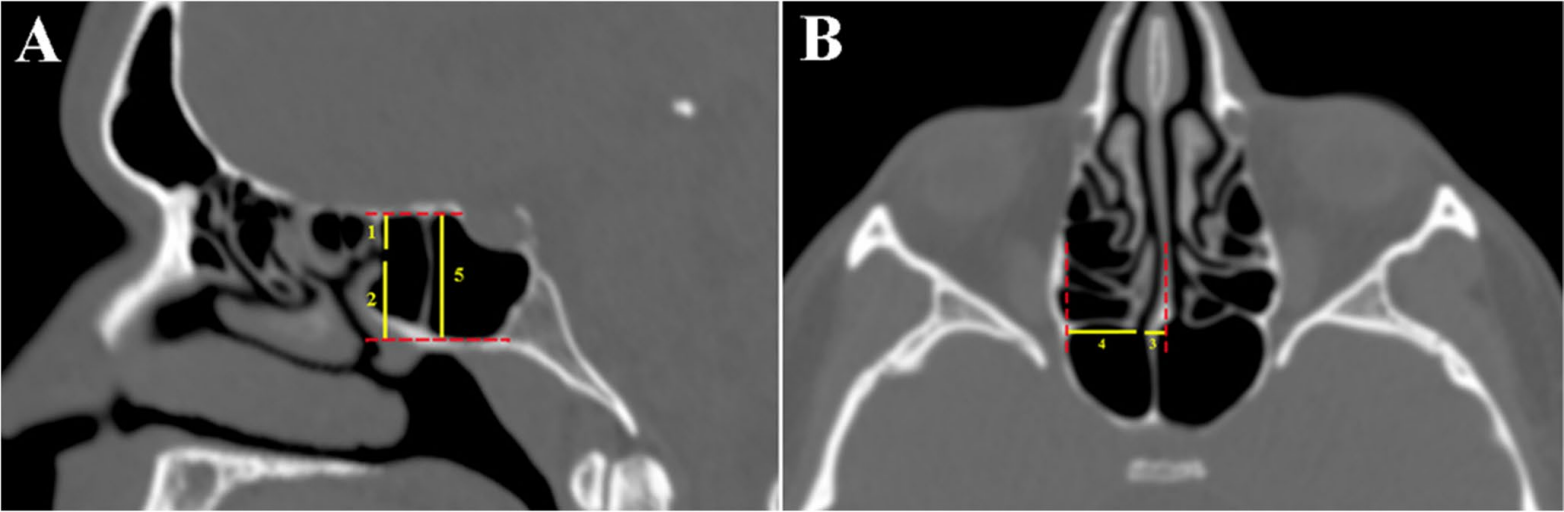
(**A**) Representative sagittal CT image showing three vertical measurements of the sphenoid sinus ostium (SSO): *Line 1*: vertical distance from the superior margin of the SSO to the roof of the sphenoid sinus. *Line 2*: vertical distance from the inferior margin of the SO to the floor of the sphenoid sinus. *Line 5*: total height of the sphenoid sinus (roof to floor). Red dotted lines indicate the superior and inferior boundaries of the sphenoid sinus, aligned with the corpus sphenoidale. (**B**) Representative axial CT image showing two horizontal measurements of the SSO: *Line 3*: distance from the medial margin of the SSO to the midline of the nasal septum (rostrum width). *Line 4*: distance from the lateral margin of the SSO to the lateral border of the anterior wall of the sphenoid sinus. Red dotted lines indicate the midline of the nasal septum and the lateral boundary of the sphenoid sinus

The vertical and horizontal locations of the SSO were assessed on sagittal and axial CT images, where the SSO was most distinctly visualized. The vertical location of the SSO was identified according to reference lines 1 and 2 on the sagittal section. Line 1 was defined as the vertical distance from the superior margin of the SSO to the roof of the SS on the sagittal section. Line 2 was defined as the vertical distance from the inferior margin of the SSO to the base of the SS on the sagittal section. Line 5, representing the sinus height, was defined as the distance from the roof to the floor of the SS (Fig. 1A).

The horizontal location of the SSO was identified using lines 3 and 4 on the axial section. Line 3, also defined as the rostrum width, was described as the horizontal distance from the medial margin of the SSO to the midline of the nasal septum. The distance between the lateral margin of the SSO and the junction of the anterior surface and lateral margin of the SS was defined as line 4 (Fig. 1B). The lateral boundary was identified as the most lateral cortical margin of the sphenoid sinus visible on the axial plane, independent of variations in pneumatisation. This definition was applied consistently across all cases.

**Fig. 2:**
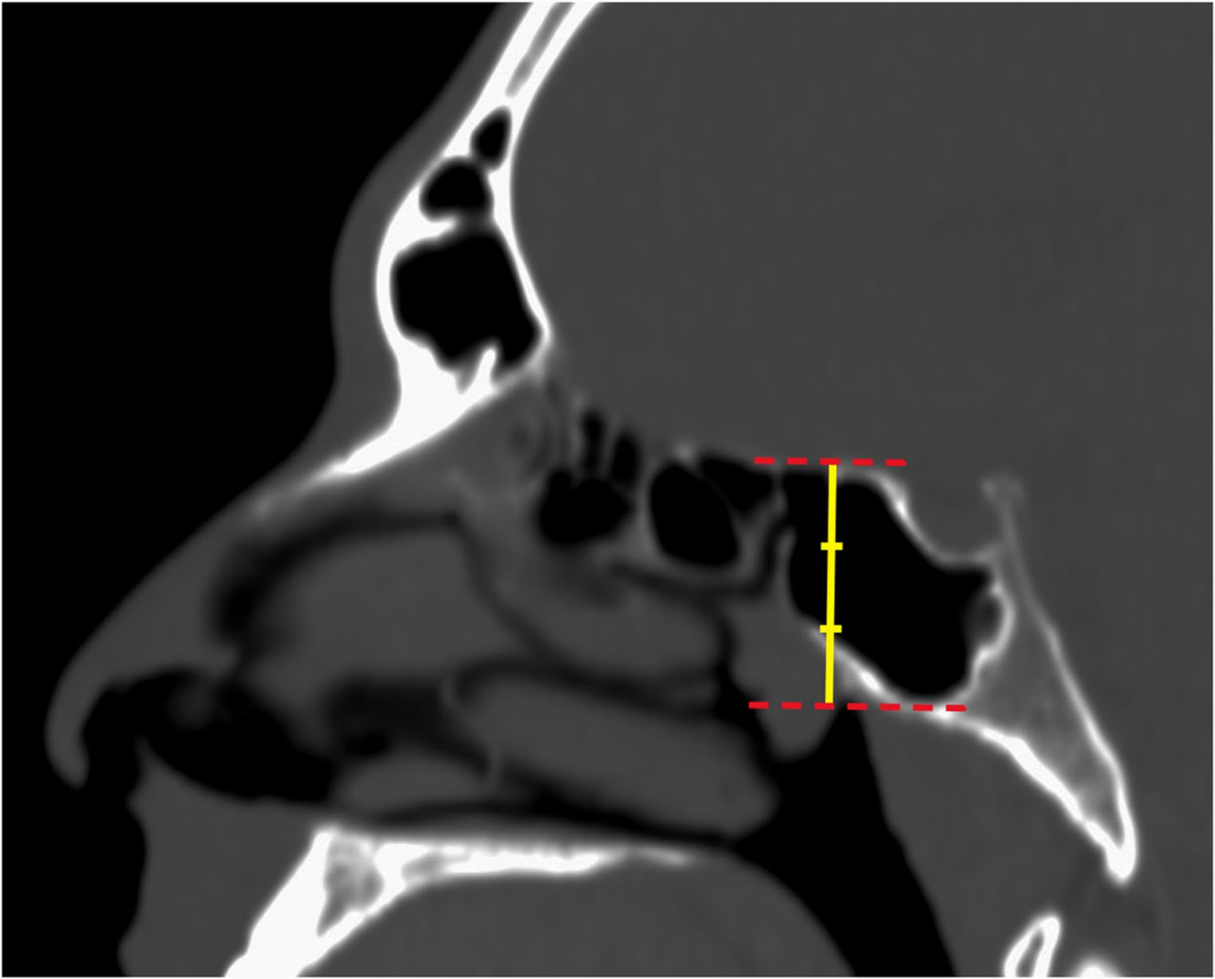
Sagittal CT image demonstrating the vertical reference line divided into three equal segments to determine the vertical position of the sphenoid sinus ostium. Red dotted lines represent the superior and inferior boundaries of the sphenoid sinus, while the yellow line indicates the vertical reference line

**Fig. 3:**
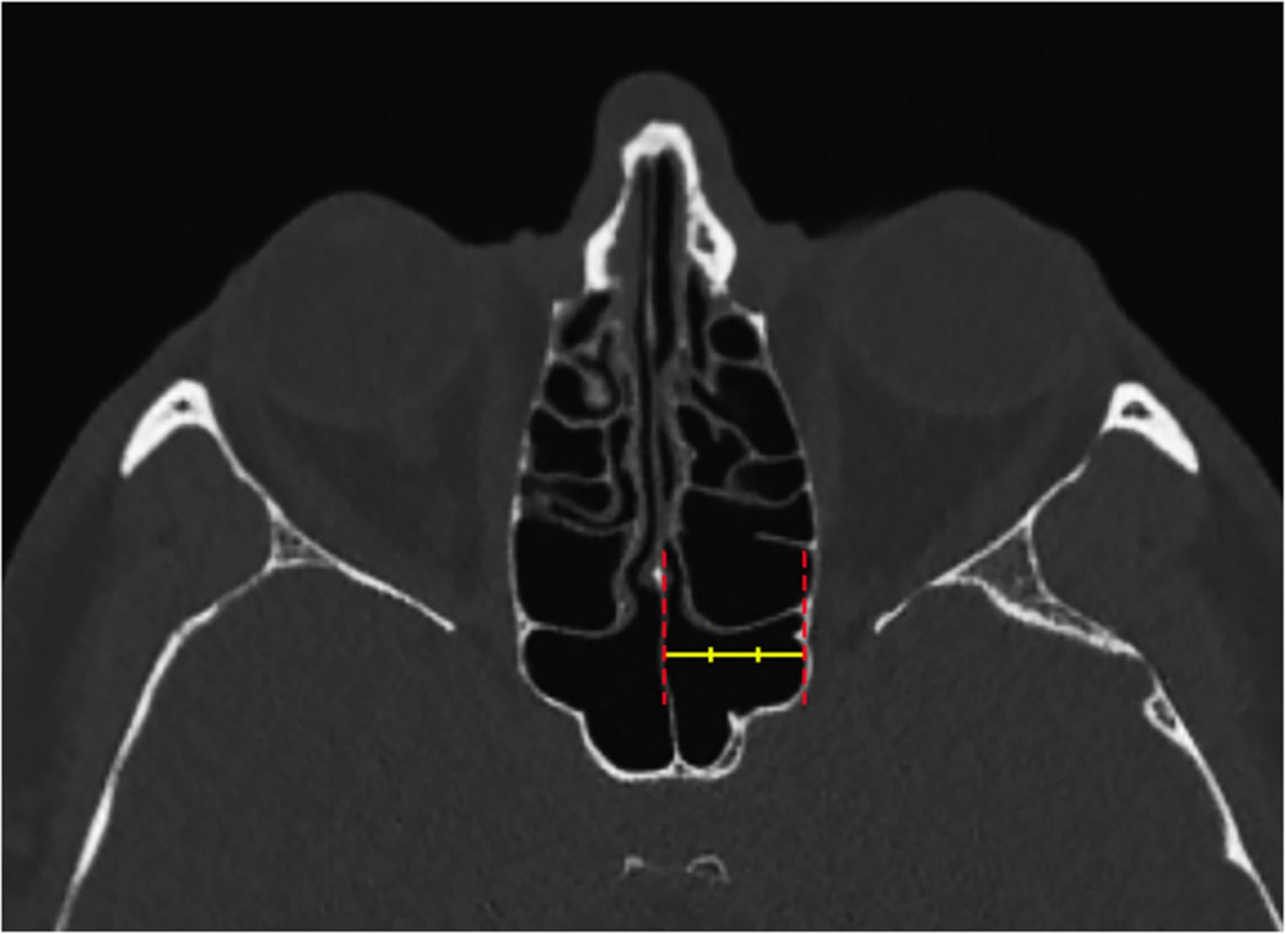
Axial CT image demonstrating the horizontal reference line divided into three equal segments to identify the horizontal position of the sphenoid sinus ostium. The yellow line represents the horizontal reference line from the medial margin of the ostium to the nasal septum. Red dotted lines indicate the midline of the nasal septum and the lateral boundary of the sphenoid sinus

The anterior wall of the SS was divided vertically into three equal parts to determine the vertical localization of the SSO. The vertical position of the SSO was categorized as upper, middle, or lower location according to where the SSO was located on the reference line (Fig. 2). In the axial sections, the distance between the reference line passing through the nasal septum and the reference line passing through the lateral boundary of the sphenoid sinus was measured and divided into three equal segments. Based on the position of the SSO along this distance, those located within the inner 1/3 were classified as medial, those within the middle 1/3 as intermediate, and those within the outer 1/3 as lateral (Fig. 3).

### Pneumatization pattern of the sphenoid sinus

**Fig. 4:**
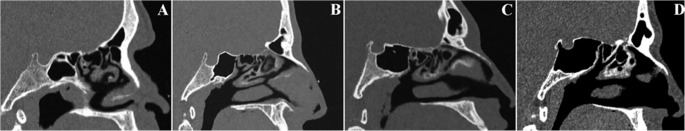
Sagittal CT images demonstrating different types of sphenoid sinus pneumatization based on the relationship of the sinus cavity to the sella turcica: (**A**) Conchal type: minimal or absent pneumatization, with no posterior extension of the sinus cavity, (**B**) Presellar type: pneumatization extends posteriorly but does not reach the sella, (**C**) Sellar type: pneumatization extends beneath the sella turcica, (**D**) Postsellar type: pneumatization extends beyond the posterior wall of the sella turcica

The degree of the SS pneumatization was determined in both sagittal and coronal planes. Sagittal CT images demonstrate different types of sphenoid sinus pneumatization based on the relationship of the sinus cavity with the sella turcica. On sagittal sections, pneumatization patterns were classified as conchal, presellar, sellar, and postsellar types, as described by Guldner et al. [10] (Fig. 4). In the conchal type, pneumatization is minimal or absent, and the sinus cavity does not extend posteriorly (Fig. 4A). In the presellar type, pneumatization extends posteriorly but does not reach the sella (Fig. 4B). In the sellar type, pneumatization extends beneath the sella turcica (Fig. 4C). In the postsellar type, pneumatization extends beyond the posterior wall of the sella turcica (Fig. 4D).

**Fig. 5:**
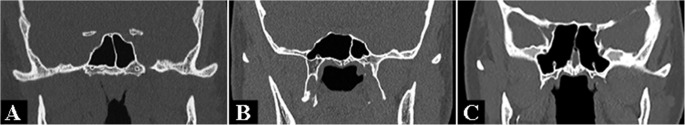
Coronal CT images illustrating the main types of sphenoid sinus pneumatization relative to adjacent neurovascular structures: (**A**) Previdian type: pneumatization extends anterior to the level of the vidian canal, (**B**) Intercanal type: pneumatization extends between the vidian canal and the foramen rotundum, (**C**) Postrotundum type: pneumatization extends posterior to the foramen rotundum

Coronal CT images demonstrate different types of sphenoid sinus pneumatization in relation to adjacent neurovascular structures. On the coronal section, pneumatization patterns were classified as previdian, intercanal, and postrotundum types (Fig. 5) [11]. In the previdian type, pneumatization extends anterior to the level of the vidian canal (Fig. 5A). In the intercanal type, pneumatization extends between the vidian canal and the foramen rotundum (Fig. 5B). In the postrotundum type, pneumatization extends posterior to the foramen rotundum (Fig. 5C).

Pneumatization of the sphenoid rostrum was defined as an air-filled, triangular bony projection extending anteriorly from the sphenoidal crest and situated within a prominent groove between the alae of the vomer. Rostrum pneumatization was considered present if the distance between the alae of the rostrum was ≥ 4 mm [12]. Coronal sections were also reviewed to determine the presence of Onodi cells. All the morphological and morphometric measurements were performed by two experienced radiologists independently, with consensus reached to minimize potential errors.

### Statistical analysis

The obtained data were analyzed using SPSS software (version 22.0, IBM SPSS Inc., Chicago, IL, USA). Categorical variables were compared using the chi-squared test or Fisher’s exact test, as appropriate. Morphometric measurements were compared according to gender and laterality using the Student’s t-test or the Mann-Whitney U test. The impact of relevant variables on the vertical and horizontal position of the SSO was assessed using binary logistic regression. Variables that were significant in the univariate regression model were subsequently included in the multivariate logistic regression model. For univariate and multivariate logistic regression analyses, the superior and medial locations of the SSO were used as reference groups in the vertical and horizontal planes, respectively, due to their higher frequency of occurrence. Similarly, sellar and previdian pneumatization types were used as reference groups for sagittal and coronal pneumatization, as they represent the most common types. For gender comparisons, the female gender was considered the reference group. To evaluate the impact of potential influencing factors on lines 1, 2, 3, and 4, multivariate linear regression analysis was performed. A value of *p* < 0.05 was considered statistically significant.

## Results

Morphometric and morphological analyses of the SS and SSO were performed on 302 CT images. The mean age of the study population was 36.44 ± 14.52 years (range, 18 to 82 years), as 37.04 ± 14.30 years for males and 35.86 ± 14.80 years for females. No significant variation was detected between the genders in terms of age (*p* = 0.618).

### Vertical location of the sphenoid sinus ostium

The SSO was most frequently located in the upper third of the vertical reference line (440 cases, 72.8%), followed by the middle third (148 cases, 24.5%) and the lower third (16 cases, 2.6%) (Table 1). The vertical localization of the SSO within the upper third of the reference line was slightly more frequent on the right side, whereas localization within the middle third was relatively more common on the left side (Table 1). A significant side-dependent variation was observed in the distribution of the vertical localization of the SSO (*p* < 0.001). On both sides, the SSO was more frequently located in the upper and lower thirds in males, while the middle third was more common in females. On the left side, the SSO was not observed in the lower third in females. No significant sex-dependent differences were found in the vertical localization of the SSO on either the right (*p* = 0.201) or left (*p* = 0.066) sides (Table 1).

**Table 1:** Distribution of the vertical and horizontal locations of the SSO according to gender and side

Location of SSO	Side	Location	Male (*n*, %)	Female (*n*, %)	Total (*n*, %)	*p* value
Vertical		**Upper**	118 (78.7%)	106 (69.7%)	224 (74.2%)	0.201
	**Right**	**Middle**	26 (17.3%)	44 (28.9%)	70 (23.2%)	
		**Lower**	6 (4.0%)	2 (1.3%)	8 (2.6%)	
		**Upper**	110 (73.3%)	106 (69.7%)	216 (71.5%)	0.066
	**Left**	**Middle**	32 (21.3%)	46 (30.3%)	78 (25.8%)	
		**Lower**	8 (5.3%)	-	8 (2.6%)	
Horizontal		**Medial**	76 (50.7%)	90 (59.2%)	166 (55.0%)	0.193
	**Right**	**Intermediate**	64 (42.7%)	60 (39.5%)	124 (41.1%)	
		**Lateral**	10 (6.7%)	2 (1.3%)	12 (4.0%)	
		**Medial**	82 (54.7%)	88 (57.39%)	170 (56.3%)	0.901
	**Left**	**Intermediate**	64 (42.7%)	60 (39.5%)	124 (41.1%)	
		**Lateral**	4 (2.7%)	4 (2.6%)	8 (2.6%)	

*SSO* Sphenoid sinus ostium, *n* number, *%* percentage, *p* < 0.05: significant difference, *p* values with asterisks are statistically significant

### Horizontal location of the sphenoid sinus ostium

The SSO was most frequently located in the medial third of the horizontal reference line (336 cases, 55.6%), followed by the intermediate third (248 cases, 41.1%) and the lateral third (20 cases, 3.3%) (Table 1). A significant side-dependent variation was observed in the distribution of the horizontal localization of the SSO (*p* < 0.001) (Table 1). The horizontal location of the SSO in the medial third was more prevalent in females, while the horizontal location of the SSO in the intermediate third and lateral third was more prevalent in males. No significant sex-dependent differences were found in the horizontal localization of the SSO on the right (*p* = 0.193) or left (*p* = 0.901) sides (Table 1).

### Pneumatization pattern of the sphenoid sinus in the sagittal plane

Of the 604 sphenoid sinus ostia, the most common type of sagittal sphenoid sinus pneumatization (SSSP) was the sellar type (478 cases, 79.1%), followed by the presellar type (68 cases, 11.3%), postsellar type (38 cases, 6.3%), and the conchal type (20 cases, 3.3%) (Table 2; Fig. 4). The incidence of the sellar type pneumatization was slightly higher on the right side, whereas postsellar and conchal types were somewhat more common on the left side. The distribution of SSSP types differed significantly between sides, indicating lateral asymmetry (*p* < 0.001). On the right side, the presellar type was more frequently observed in females, whereas the postsellar type was more common in males. On the left side, the sellar type was more often seen in females, while the postsellar type was more frequently identified in males. The prevalence of SSSP types did not significantly differ between genders on the right (*p* = 0.097) or left (*p* = 0.498) sides (Table 2).

**Table 2:** Distribution of types of sagittal and coronal sphenoid sinus pneumatization according to gender and side

	Side	Pneumatization type	Male (n, %)	Female (n, %)	Total (n, %)	*p* value
SSSP	**Right**	Sellar	120 (80.8)	124 (81.6)	244 (80.8)	0.097
		Presellar	12 (8.0)	22 (14.5)	34 (11.3)	
		Postsellar	14 (9.3)	2 (1.3)	16 (5.3)	
		Conchal	4 (2.7)	4 (2.6)	8 (2.6)	
	**Left**	Sellar	112 (74.4)	122 (80.7)	234 (77.5)	0.498
		Presellar	16 (10.7)	18 (11.8)	34 (11.3)	
		Postsellar	16 (10.7)	6 (3.9)	22 (7.3)	
		Conchal	6 (4.0)	6 (3.9)	12 (4.0)	
CSSP		Previdian	86 (57.3)	84 (55.3)	170 (56.3)	0.863
	**Right**	Intercanal	32 (21.3)	38 (25.0)	70 (23.2)	
		Postrotundum	32 (21.3)	30 (19.7)	62 (20.5)	
		Previdian	76 (50.7)	80 (52.6)	156 (51.7)	0.908
	**Left**	Intercanal	34 (22.7)	30 (19.7)	64 (21.2)	
		Postrotundum	40 (26,7)	42 (27.6)	82 (27.2)	

*SSSP* Sagittal sphenoid sinus pneumatization, *CSSP* Coronal sphenoid sinus pneumatization *n* number, *%* percentage, *p* < 0.05: significant difference, *p* values with asterisks are statistically significant

### Pneumatization pattern of the sphenoid sinus in the coronal plane

The prevalence of coronal sphenoid sinus pneumatization (CSSP), ranked from most to least common, was identified as the previdian type (326 cases, 54%), postrotundum type (144 cases, 23.8%), and intercanal type (134 cases, 22.2%) (Table 2; Fig. 5). The previdian type was more common on the right side, while postrotundum type was more frequently observed on the left side (Table 2). A significant variation in the incidence of CSSP types was identified based on laterality (*p* < 0.001). The distribution of CSSP types did not differ significantly between males and females on either the right (*p* = 0.863) or left (*p* = 0.908) sides, indicating no sex-based variation (Table 2).

### Prevalence of rostrum pneumatization and Onodi cells

Rostrum pneumatization was identified in 62 cases (41.1%), comprising 29 males (38.7%) and 33 females (43.4%). No significant sex-related difference was observed (*p* = 0.553). Onodi cells were present in 112 cases (18.5%), comprising 44 males (14.7%) and 68 females (22.4%). Of these, 60 were identified on the right side and 52 on the left side. No significant differences in the presence of Onodi cells were found with respect to sex or laterality (*p* = 0.223 and *p* > 0.05, respectively).

### Morphometric characteristics of the sphenoid sinus and sphenoid sinus ostium

The comparisons of morphometric measurements of SSO and SS according to gender and laterality are presented in Table 3. The mean length of line 1 was 9.46 ± 2.99 mm on the right side and 9.48 ± 2.96 mm on the left side, with no significant difference between the sides (*p* = 0.954). Although the mean length of line 1 was greater in females than in males, no significant gender-related differences were found on the right (*p* = 0.233) or left (*p* = 0.950) sides (Table 3). The mean length of line 2 was 20.72 ± 3.64 mm on the right side and 20.47 ± 4.13 mm on the left side, with no right and left-dependent differences (*p* = 0.661). Males had significantly longer line 2 measurements on both sides compared with females (*p* = 0.001 and *p* = 0.006, respectively) (Table 3). The mean length of line 3 was 3.06 ± 1.60 mm on the right side and 2.90 ± 1.57 mm on the left side, with no statistically significant difference between the sides (*p* = 0.260). No significant gender-related differences were observed in the mean length of line 3 on the right (*p* = 0.387) and left sides (*p* = 0.266). A significant difference was observed in the mean length of line 4 between the right and left sides (10.45 ± 2.35 mm vs. 9.95 ± 2.18 mm, respectively, *p* = 0.039). On the right side, line 4 was significantly longer in males compared with females (*p* = 0.037), whereas no significant gender-related difference was detected on the left side (*p* = 0.068) (Table 3). The mean length of line 5 was 22.81 ± 4.25 mm on the right and 22.50 ± 4.55 mm on the left side, with no significant differences between the sides (*p* = 0.537). Line 5 was significantly longer in males on both sides compared with females (*p* = 0.001 and *p* = 0.006, respectively) (Table 3).

**Table 3:** Comparison of vertical and horizontal distance measurements of the sphenoid sinus ostium according to gender and side

Parameter	Side	Male	Female	*p* value
Line 1	Right	9.17 ± 2.88	9.75 ± 3.08	0.233
	Left	9.47 ± 2.92	9.50 ± 3.01	0.950
Line 2	Right	21.27 ± 3.86	19.29 ± 3.14	0.001*
	Left	21.38 ± 4.45	19.56 ± 3.58	0.006*
Line 3	Right	2.90 ± 1.71	3.22 ± 1.47	0.387
	Left	2.78 ± 1.65	3.02 ± 1.48	0.266
Line 4	Right	10.85 ± 2.39	10.05 ± 2.25	0.037*
	Left	10.28 ± 2.44	9.63 ± 1.84	0.068
Line 5	Right	23.95 ± 4.59	21.69 ± 3.58	0.001*
	Left	23.51 ± 4.86	21.49 ± 4.01	0.006*

Data expressed in mm and mean ± standard deviation, *p* values with asterisks are statistically significant

### Effect of the potential factors on the vertical location of the sphenoid sinus ostium

The variables affecting the vertical location of the SSO are presented in Table 4. A strong positive association was observed between the upper location of the SSO and the postrotundum type of the SS. Compared with the previdian type, the postrotundum type increased the probability of the SSO being in the upper location by 3.289-fold (OR 3.289, *p* = 0.019, 95% CI: 1.216–8.897). A significant positive relationship was also found between rostrum width and the upper location of the SSO. Each unit increase in rostrum width increased the likelihood of the SSO being located in the upper location by 1.277-fold (OR 1.277, *p* = 0.047, 95% CI: 1.003–1.625) (Table 4).

**Table 4:** Results of multivariate logistic regression analysis to determine the effect of potential factors on the upper location of the sphenoid sinus ostium

Variable	B	SE	95% CI	OR	*p* value
Gender (Female)	0.047	0.383	0.495–2.218	1.048	0.903
Age	-0.012	0.013	0.963–1.013	0.988	0.345
CSSP (Postrotundum)	1.191	0.508	1.216–8.897	3.289	0.019*
Rostrum width	0.244	0.123	1.003–1.625	1.277	0.047*

*CSSP* coronal sphenoid sinus pneumatization, *B* regression coefficient, *SE* standard error, *OR* odds ratio, *CI* confidence interval, *p* < 0.05: significant difference, *p* values with asterisks are statistically significant

The variables affecting the vertical distance between the superior margin of the SSO and the roof of the SS (Line 1) are shown in Table 5. A significant inverse association was found between the rostrum pneumatization and the length of line 1. Rostrum pneumatization shortened line 1, shifting the SSO position superiorly by 0.627 units. (B: -0.627, *p* = 0.013, 95% CI: -1.122 – -0.132). The presence of Onodi cells and the length of line 5 were positively and significantly associated with the length of line 1. The presence of Onodi cells shifted the SSO position inferiorly by 1.929 units (B: 1.929, *p* < 0.001, 95% CI: 1.293–2.565). Similarly, each unit increase in the length of line 5 shifted the vertical position of the SSO towards the inferior border by 0.341 units (B: 0.341, *p* < 0.001, 95% CI: 0.274–0.408) (Table 5).

**Table 5:** Results of multivariate linear regression analysis to determine the effect of the potential factors on the vertical distance of the ostium to the roof of the sphenoid sinus (Line 1)

Variable	B	SE	95% CI	*p* value
Gender (Female)	2.243	0.249	-0.247–0.733	0.330
Age	-0.012	0.009	-0.028–0.005	0.178
Rostrum pneumatization	-0.627	0.252	-1.112 – -0.132	0.013*
Onodi cell	1.929	0.323	1.293–2.565	< 0.001*
Line 5	0.341	0.034	0.274–0.408	< 0.001*

*SSSP* sagittal sphenoid sinus pneumatization, *B* regression coefficient, *SE* standard error, *CI* confidence interval, *p* < 0.05: significant difference, *p* values with asterisks are statistically significant

### Effect of the potential factors on the horizontal location of the sphenoid sinus ostium

The variables affecting the horizontal location of the SSO are presented in Table 6. A significant positive association was identified between gender, the conchal type, and the postrotundum type with the medial location of the SSO. In females, the probability of the SSO being in the medial location was 2.715-fold higher than in males (OR: 2.715, *p* = 0.007, 95% CI: 1.307–5.643). In the conchal type, the likelihood of the SSO being in the medial location increased 9.820-fold compared with the sellar type (OR: 9.820, *p* = 0.031, 95% CI: 1.228–78.521). Compared with the previdian type, the probability of medial SSO localization increased 2.978-fold in the postrotundum type (OR: 2.978, *p* = 0.028, 95% CI: 1.125–7.881).

**Table 6:** Results of multivariate logistic regression analysis to determine the effect of potential factors on the medial location of the sphenoid sinus ostium

Variable	B	SE	95% CI	OR	*p* value
Gender (Female)	0.999	0.373	1.307–5.643	2.715	0.007*
Age	0.014	0.013	0.987–1.041	1.014	0.305
SSSP (Postsellar)	-1.570	0.802	0.043–1.001	0.208	0.049*
SSSP (Conchal)	2.284	1.061	1.228–78.521	9.820	0.031*
CSSP (Postrotundum)	1.091	0.497	1.125–7.881	2.978	0.028*
Rostrum width	-1.762	0.193	0.118–0.251	0.172	< 0.001*

*SSSP* sagittal sphenoid sinus pneumatization, *CSSP* coronal sphenoid sinus pneumatization, *B* regression coefficient, *SE* standard error, *OR* odds ratio, *CI* confidence interval, *p* < 0.05: significant difference, *p* values with asterisks are statistically significant

A negative association was found between the postsellar type and rostrum width with the medial location of the SSO. Compared with the sellar type, the postsellar type reduced the likelihood of a medial SSO location 0.208-fold (OR: 0.208, *p* = 0.049, 95% CI: 0.043–1.001). In individuals with a wider rostrum, medial SSO localization was reduced 0.172-fold (OR: 0.172, *p* < 0.001, 95% CI: 0.118–0.251).

The variables affecting the horizontal measurement between the medial margin of the SSO and the nasal septum (line 3) are shown in Table 7. A significant inverse association was found between gender and the length of line 3. In females, compared with males, line 3 tended to be 0.444 units shorter, indicating a trend of medialization in the SSO. (B:-0.444, *p* < 0.001, 95% CI: -0.679, -0.208) (Table 7).

**Table 7:** Results of multivariate linear regression analysis to determine the effect of potential factors on horizontal distance of the ostium to the nasal septum (Line 3)

Variable	SE	B	95% CI	*p* value
Gender (Female)	0.120	-0.444	-0.679 – -0.208	< 0.001*
Age	0.004	0.004	-0.004–0.012	0.312

*B* regression coefficient, *SE* standard error, *CI* confidence interval, *p* < 0.05: significant difference, *p* values with asterisks are statistically significant

## Discussion

In the present study, analyses were made of the vertical and horizontal positions of the SSO, and the influence of factors such as age, gender, and sagittal or coronal pneumatization types of the SS. The vertical and horizontal positions of the SSO were evaluated in both sagittal and horizontal sections using lines 1, 2, 3, and 4, which have been described and standardized in previous studies, according to sex and laterality [3, 9].

When the vertical and horizontal distances of the SSO were evaluated according to gender, the current study findings revealed that the measurements were generally greater in males, with significantly higher values for lines 2 and 5 on both sides and line 4 on the right side. These sex-dependent differences may be attributed to the greater degree of SS pneumatization observed in males compared with females, as reported in previous studies [13–15]. The data also indicated symmetry in the overall localization of the SSO, except for line 4 measurements. The SSO was located slightly more laterally on the left side than on the right. This cranial symmetry in SSO localization is consistent with previous reports by Kim et al., Halawi et al., and Wu et al. [9, 16, 17].

Unlike the previous studies by Kim et al. (2023) and Halawi et al. (2015), the novelty of our research lies in its comprehensive assessment of the positional variability of the SSO using a larger radiological dataset (302 CT scans, 604 sides in total) [9, 16]. By incorporating multiple potential determinants through multivariate linear and logistic regression analyses, we demonstrated that several factors, including gender, rostrum width, and the sagittal and coronal pneumatization patterns of the sphenoid sinus, significantly influence the horizontal and vertical positioning of the SSO. According to multivariate logistic regression analysis, the tendency of the SSO to be in the medial location was 2.715-fold greater in females, compared to males. In other words, males were more likely to have the SSO located in the intermediate or lateral portion. In addition, line 3 was 0.444 units shorter in females, indicating a tendency toward medialization of the SSO compared with males. Such a sex-specific effect on SSO localization has not been previously reported. This study is the first to report a strong effect of sex on the horizontal position of the SSO. This sex-related difference may be explained by variations in cranial morphology, SS size, or the degree of sinus pneumatization between sexes. Sexual dimorphism in craniofacial morphology may explain this variation: females generally have a smaller, more compact sinonasal structure, favoring medial SSO placement, whereas males tend to have a larger, more pneumatized sphenoid sinus, shifting the SSO away from the medial third [18, 19]. Ethnic variations should also be considered. Recognition of sex-specific positional tendencies of the SSO may provide valuable guidance for surgeons in selecting the most appropriate entry point for transsphenoidal interventions.

When the effect of coronal pneumatization on the SSO location was analyzed, a two-way influence was observed. Compared with the previdian type, the postrotundum type increased the probability of the SSO being in the medial position by 2.978-fold and in the superior position by 3.289-fold. Accordingly, in the previdian type, the SSO was more frequently located in the lateral and inferior thirds than in the postrotundum type. Similarly, Doubi et al. reported that coronal SS pneumatization shifts the SSO laterally [3]. However, in a recent study by Kim et al., no significant impact of the coronal pneumatization types on the horizontal position of the natural SSO was suggested [9]. To date, the potential effect of coronal pneumatization on the vertical position of the SSO has not been reported. Notably, the current study findings indicate that coronal pneumatization may shift the SSO location superiorly or inferiorly depending on the pneumatization pattern, suggesting that surgeons should consider individual anatomic variations to optimize the approach and minimize intraoperative complications.

The influence of sagittal sphenoid sinus pneumatization on SSO location has been previously documented. Kim et al. reported that the probability of the SSO being closer to the medial border increased 0.223-fold in the postsellar type compared with the presellar type, but no association was found for the sellar type [9]. Likewise, Halawi et al. observed that the natural SSO was positioned closer to the midline in the sellar and presellar types than in the postsellar type [16]. In the present study, the conchal type was identified as a major influencing factor, increasing the likelihood of a medial SSO position 9.82-fold compared with the sellar type. Interestingly, the postsellar type, when compared with the sellar type, showed an inverse effect, reducing the probability of a medial location by 0.208-fold. These findings suggest that different pneumatization types may have distinct effects on the horizontal location of the SSO. Therefore, detailed preoperative evaluation of sagittal pneumatization patterns on CT may allow prediction of medialization or lateralization of the SO, thereby contributing to safer surgical planning.

Partial or complete resection of the sphenoidal rostrum, a triangular bony protrusion on the anterior surface of the sphenoid body, is a routine procedure to facilitate access to pituitary tumors [20]. An increased tendency for the SSO to be located in the middle or lateral portion, rather than the medial portion, in the presence of a well-developed rostrum has been previously reported [9]. The current study findings support this observation, as the data demonstrated that the probability of the SSO being located medially was 0.172-fold lower in cases with a wider rostrum. Notably, when the natural SSO is positioned more laterally, the surgical approach may become more challenging; therefore, careful preoperative planning is essential. Interestingly, the current study showed that a prominent rostrum also significantly influences the vertical location of the SSO, increasing the tendency of the SSO to be in the upper position by 1.277-fold. Thus, similar to the two-way effect of the postrotundum type, a well-developed rostrum also affects both horizontal and vertical positions of the SSO. Accordingly, the presence of a well-developed rostrum on preoperative CT imaging may serve as a reliable predictor of the superior and lateral localization of the SSO, which should be carefully considered during surgical planning.

The findings of this study also revealed that the SSO was positioned 0.627 units closer to the superior border in the presence of rostrum pneumatization. To date, no study has reported the potential influence of rostrum pneumatization on the vertical localization of the SSO. Doubi et al. suggested that the SSO is closer to the lateral border in cases with a pneumatized rostrum, and stated that the SSO was more commonly situated in the middle portion in such cases [3]. However, in the current study, no significant relationship was found between rostrum pneumatization and the horizontal localization of the SSO. The Onodi cell is an important anatomic variant due to its close relationship with the optic nerve and internal carotid artery. The presence of an Onodi cell can increase the risk of injury to these structures during functional SS surgery and may even be mistaken for the SS itself [21, 22].

In the current study, the presence of Onodi cells was seen to increase the distance between the SSO and the roof of the sinus, resulting in the SSO being positioned 1.929 units closer to the sinus base. This finding is consistent with the studies of Kim et al. and Hwang et al., which reported that the SSO was located more inferiorly in the presence of Onodi cells [9, 23]. These findings suggest that Onodi cells may displace the sinus roof downward, thereby shifting the SSO inferiorly. In the present study, sinus height was found to influence the vertical position of the SSO. Specifically, the SSO tended to be located more inferiorly in larger SS. Each unit increase in sinus height shifted the vertical position of the SSO toward the inferior border by 0.341 units. This relationship between SSO position and sinus height is consistent with the findings of Doubi et al. and Hidir et al. [3, 24]. Considering the potential influence of sinus height on SSO location, the relatively inferior position of the SSO observed in males compared with females may be attributed to the generally greater sphenoid sinus height in males.

Some limitations of the study should be considered, primarily the retrospective design, which may have introduced selection bias. Paranasal CT images of individuals who were admitted to the hospital for various reasons, such as falling off a bicycle, head trauma, headache, seizure, high fever, and dizziness, were included in the study. In the electronic medical record system of the hospital, none of the patients included in our study had any diagnosed sinonasal pathology, nor had they undergone any surgical or medical treatment related to the paranasal region. Only CT scans of individuals who were discharged without receiving any specific treatment after clinical evaluation were included. Although our study population consists of individuals who underwent CT imaging for the reasons mentioned above, we believe that this does not introduce selection bias that would compromise the validity of our findings. This is because, in our institution, paranasal CT imaging is not performed on healthy volunteers. Therefore, by the nature of retrospective imaging-based studies, our study sample cannot include volunteer participants. Secondly, this study consisted of healthy subjects only, so it does not include clinical outcomes, thereby preventing direct clinical correlation between the observed variations and surgical complications or patient prognosis. Further investigations, including subjects with various SS pathologies, are needed to confirm the potential influence of these factors on the SSO localization. Nonetheless, the results of this study provide a basis for future research analyzing the impact of SS morphology and its surrounding structures on the position of the SSO in patients with SS pathologies.

## Conclusion

The findings of this study highlight that the localization of the SSO can be influenced by multiple factors to different degrees. Gender was seen to be a major determinant causing medialization of the SSO in females and lateralization of the SSO in males. The probability of the SSO being located medially increased in the conchal type, while this probability was reduced by the postsellar type. Post-rotundum type pneumatization and the presence of a well-developed rostrum exhibited a two-way effect on SSO location. Compared with the previdian type, the postrotundum type increased the tendency for medial SSO location. In contrast, a wider rostrum had the opposite effect, increasing the likelihood of the SSO being located laterally. In addition, the presence of a postrotundum type and a highly developed rostrum increased the probability of the SSO being located superiorly, whereas the presence of Onodi cells and greater sinus height shifted the SSO closer to the inferior border. Careful preoperative evaluation of CT images for these potential factors may therefore provide valuable guidance to surgeons and help minimize the risk of serious complications during endoscopic sinus surgery.

## Data Availability

All data for this study is presented in this paper.
